# The Burden of Out-of-Pocket Expenditure for Emergency Health Care Service in St. Paul Hospital Millennium Medical College, Addis Ababa Ethiopia

**DOI:** 10.4314/ejhs.v35i2.11

**Published:** 2025-03

**Authors:** Woldesenbet Waganew

**Affiliations:** 1 St Paul hospital millennium medical college

**Keywords:** out of pocket payment, emergency service, health care expenditure

## Abstract

**Background:**

High burden of out-of-pocket health care expenditure is a known factor that affects universal access health care, in general and acute care more specifically. The aim of this study was to assess the burden of out-of-pocket payment in acutely ill patients in emergency department.

**Methods:**

A cross-sectional study was conducted by reviewing patient record visited St. Paul Hospital Millennium Medical College, Addis Ababa, Ethiopia in study period. Data were analysed using SPSS Version 25. Descriptive statistics was used to summarize the finding.

**Results:**

A total of 22,982 clients were seen, with 388 eligible charts examined. Participants ranged in age from 15 to 100 years, with a mean age of 49.84 ± 18.21 years. The male-to-female ratio among participants was 1.3:1. Most participants were from Addis Ababa (52.8%), followed by Oromia (31.4%). Among the specified payment methods, out-of-pocket payments were the most prevalent at 35.8%, followed by community-based health insurance at 15.5% and government fee waivers at 13.7%.

**Conclusion:**

Out-of-pocket payments were the predominant method of payment, followed by Community-Based Health Insurance (CBHI) and government waivers. Out-of-pocket spending consistently exceeded other payment methods across all age groups, both genders, various geographic regions, referral sources, and among individuals with comorbid conditions.

## Introduction

WHO defines universal health coverage (UHC) as a society having all needed service of high quality without cost of payment for service being a barrier. Following the WHO report of 2010, countries reform their health financing strategy in relation to UHC. Its fact that aligned strategy is key in achieving UHC. Health finance strategy of the nation is believed to be a rate limiting factor for achieving UHC, which in turn is affected by: revenue raising, risk pooling and purchasing in the context of equity resource distribution, efficiency, transparency and accountability being owned and oversighted by the government. This concept targeted quality, equity and financial protection (1-9). Out of pocket (OOP) is a global health problem in about 70 countries where it remains more than 20% and exceeds 35% in Sub Saharan Africa (10).

The aim of the Ethiopian government is to achieve universal health coverage in 2030. Despite implantation of different health care financing reforms, the system is being challenged with high-out of pocket expenditure. To hasten the transition to UHC, health financing policy should follow the guiding concepts of coercion and subsidization. There wasn't a single health financing strategy that worked well in all nations. However, the optimal course of action for one country in a certain situation could not be appropriate for another one. The Ethiopian government unveiled a 20-year strategy in 2015 to attain UHC. The three focal points of Ethiopia's current health policy for UHC, among other things, are the creation of a fair and acceptable standard of health care, assurance of universal access to care, and supply of care under a system of payment with unique aid mechanisms (11, 12).

Out of pocket payments are not a better way to obtain equitable healthcare without suffering financial hardship; community-based health insurance (CBHI) and social health insurance (SHI) are preferable options. Ethiopia's healthcare system is financed by a variety of sources, including out-of-pocket (OOP) payments (35.8%), the Ethiopian government (16.5%), loans and donations from all over the world (46.8%), and others (0.9%). (13, 14).

Understanding the pattern and statistics of health care financing at service provider level is important scientific information that should be available for the constant evaluation of health services and continuous quality improvement and informative in achieving universal health care. The trend of method of payment statistics is crucial scientific data that has to be accessible for ongoing service accessibility and affordability assessment and quality improvement. There is currently a paucity of information on health care expenditure at health facility level.

This study aims to assess the burden and predictive factors of out-of-pocket payment expenditure among patients presenting to emergency departments. This study may offer evidence for quality improvement, and provides comparison data and baseline for health care financing. Additionally, could be scientific evidence for hospital administrators, policy makers, and the large scientific community in general.

## Materials and Methods

**Study setting and period**: The study was conducted from January 1, 2020- June 30, 2022 in St. Paul Hospital millennium medical college (SPMMC)-ED. SPMMC is one of the biggest hospitals in Ethiopia, providing tertiary level referral treatment. The Hospital is open 24/7 for emergency services. The hospital provides offers diagnostic testing and treatment for approximately 370,000-400,000 patients per year (15).

**Study design and population**: A cross-sectional study design was used. The study population were participants who had been served in SPHMMC-ED in study period. All patients with charts in ED patients during study period were reviewed. Patients' charts with no triage paper, age below 12 were excluded.

**Sample size and sampling**: Since there is no research done in the same set up the sample size was estimated using single population proportion with the assumption of 50% minimum prevalence of triage format completeness patients, and level of confidence 95%, 5 % margin of error it yields a sample of 385 cards. Patients with charts were selected using convenient sampling techniques.

**Data collection procedures, and management**: For the purpose of this study, potential charts were traced using patients name and hospital's given identification numbers from ED triage logbook. Traceable charts with complete information were reviewed, in addition data has been cross checked with finance logbook for method of payment. Data were abstracted by trained research assistants with a minimum of BSC in health-related fields who took training specifically on this tool. Data had been collected with chart review. Prior to enrolment, all research staff participated in human subjects' protection training to ensure data confidentiality for all study participants. Local staff were involved as facilitators were trained and won human subjects in line with Ethiopian Ministry of health standards. The consultant ensured compliance with ethics by site visits and ongoing training and communication. Random checks by implementation staff on completion of data forms and adherence to the protocol were made by consultants.

**Statistical analysis**: Descriptive statistics were computed using statistical software package (SPSS). Frequency distributions were generated for categorical variables to illustrate the proportion of different categories within the dataset.

**The following operational definitions were used**:

*Out-of-pocket payment*: an expense clients made for emergency health care services with their own money.

*Principal diagnosis*: an assumed primary diagnosis/es for which patient get treated in ED.

*Co-morbidity*: underlying chronic illness that is not directly associated with the main causes that made clients to visit ED.

**Ethical considerations**: Letter of ethical clearance was obtained from St. Paul Hospital millennium medical college IRB. The study has been conducted in compliance with Ethiopian national and regional regulations and guidelines applicable to research involving human subjects. Prior to study enrolment, all research staff have been trained in principles of human subjects' protection and management of confidential study materials. Data were not collected directly from patients. No vulnerable populations directly participated in the study. Data from questionnaires and study materials entered into electronic databases. Both paper study forms and electronic database data had no identifying information. All study forms were identified with only the study ID. There likely were no direct benefit for participating in this study. Participants were not compensated for completing the study.

## Results

The study findings are presented in four subcategories for logical understanding as: sociodemographic characteristics (age, sex, address region), pre-hospital characteristics (source of referral, and mode of arrival to ED), and clinical details (frequency and distribution of working diagnosis in ED, comorbid conditions), health care financing details and binary and multivariate logistic regression findings.

**Socio-demography characteristics**: During the 30-months study period, 22,982 adult critically ill patients visited the ED. Traceable 693 ED-served patients' charts were identified; 53 of them were lost, 388 qualified for analysis, and the remaining 252 charts (36.36%) were excluded due to incomplete information.

The age range was from 15 to 100 with a mean age of 49.84 ± 18.21 years with most common participants being in age group between 30-60, 185 (47.7%) followed by age group more than or equal to 60, 135 (34.8%) and age group less than or equal to 30 years, 68 (17.5%). Male to female ratio of the participants was 1.3:1. The majority of the participants came from Addis Ababa region (52.8%) followed by Oromia (31.4%), and the least being participants were from Gambella region 1 (0.3%). In 3.9% of participants the address region is unknown (Table 1).

**Table 1:** Method of payment in cross tabulation with Socio-demographic, pre-hospital, and clinical characteristics of study participants in SPHMMC-ED from January 1 2020 to June 30 2022 Addis Ababa; Ethiopia

Variables		Fee waived by government N (%)* 54(13.9%)	Out of pocket N (%) 139 (35.8%)	CBHI N (%)* 60 (15.5%)	Unspecified method of payment N (%)* 135 (34.8%)
Age in years	≤30 N% 68(17.5%)	16 (23.5%)	26 (38.2%)	8 (11.8%)	18 (26.5%)
	30-60 N% 185 (47.7%)	25 (13.5%)	61 (33.0%)	26 (14.1%)	73 (39.5%)
	≥60 N% 135(34.8%)	13 (9.6%)	52 (38.5%)	26 (19.3%)	44 (32.6%)
Sex	Male N% 221(56.9%)	27 (12.2%)	72 (32.6%)	36 (16.3%)	86 (38.9%)
	Female N% 167(43.1%)	27 (16.2%)	67 (40.1%)	24 (14.4%)	49 (29.3%)
Address region	Addis Ababa N% 205(52.8%)	29 (14.1%)	66 (32.2%)	38 (18.5%)	72 (35.1%)
	Oromia N% 122 (31.4%)	19 (15.6%)	46 (37.7%)	14 (11.5%)	43 (35.2%)
	Amhara N% 15(3.8%)	1 (6.7%)	7 (46.7%)	2 (13.3%)	5 (33.3%)
	SNNPR N% 15 (3.8%)	1 (6.7%)	6 (40.0%)	3 (20.0%)	5 (33.3)
	Benishangul Gumuz N% 5(1.3%)	1 (20%)	3 (60%)	0 (0%)	1(20%)
	Gambela N% 2(0.5%)	0 (0%)	1(100%)	0(0%)	0(0%)
	Dire Dawa N% 2 (0.5%)	0 (0%)	1(50%)	0 (0%)	1 (50%)
	Afar N% 4(1.0%)	1 (25%)	2 (50%)	0(0%)	1(25%)
	Tigray N% 2(0.5%)	1 (50%)	1 (50%)	0(0%)	0(0%)
	Sidama N% 2 (0.5%)	0 (0%)	0 (0%)	2 (100%)	0 (0%)
	Other N%13 (3,3%)	1 (7.69%)	6 (46.15%)	1 (7.69%)	7 (53.84%)
Source of Referral	Public Hospital N% 87(22.4%)	10 (11.5%)	39 (44.8%)	9 (10.3%)	29 (33.3%)
	Health center N% 54(13.9%)	9 (16.7%)	16 (29.6%)	16 (29.6%)	13 (24.1%)
	Private Hospital N% 25 (6.4%)	1 (4%)	11 (44.0%)	4 (16.0%)	9 (36.0%)
	Private clinic N% 2 (0.5%)	0 (0%)	1 (50%)	0 (0%)	1 (50%)
	Self-referred N% 118 (30.4%)	14 (11.9%)	38 (32.2%)	16 (13.6%)	50 (42.4%)
	Unknown N% 52(13.4%%)	5 (9.6%)	19 (36.5%)	3(5.8%)	25 (48.1%)
	Sent from SPHMMC-OPD N% 42(10.8%%)	8 (19.0%)	15 (35.7%)	12 (28.6%)	7 (16.7%)
	Other N% 8(2.1%%)	7 (87.5%)	0 (0%)	0 (0%)	1 (12.5%)
Mode of arrival	Ambulance N% 74(19.0%)	15 (20.3%)	23 (31.1%)	11 (14.9%)	25 (33.8%)
	Taxi N% 90(23.2%)	14 (15.6%)	38 (42.2%)	14 (15.6%)	24 (26.7%)
	private car N% 68(17.5%)	9 (13.2%)	22 (32.4%)	10 (14.7%)	27 (39.7%)
	Others N% 17(4.3%)	4 (23.5%)	4 (23.5%)	4 (23.5%)	5 (29.4%)
	Unknown N% 61(15.7%)	7 (11.5%)	17 (27.9%)	6 (9.8%)	31 (50.8%)
	public transport N% 45(11.6%)	2 (4.4%)	21 (46.7%)	7 (15.6%)	15 (33.3%)
	Walking N% 33(8.5%)	3 (9.1%)	14 (42.4%)	8 (24.2%)	8 (24.2%)
Comorbid illness	Renal related diseases N% 49(12.6%)	8 (16.3%)	16 (32.7%)	9 (18.4%)	16 (32.7%))
	Malignancy N% 49(12.6%)	9 (18.4)	21 (42.9%)	5 (10.2%)	14(28.6%)
	Hypertension N% 43(11.1%),	4 (9.3%)	20 (46.6%)	8 (18.6%)	11(25.6%)
	Respiratory illness N% 29 (7.5%),	6 (25%)	9 (37.5%)	3 (12.5%)	6 (25%)
	Other CVD N %27 (7.0%)	6 (22.2%)	9 (33.3%)	6 (22.2%)	6 (22.2%)
	DM/its complications N% 25 (6.4%)	4 (16.0%)	4 (16.0%)	4 (16.0%)	13 (52.0%)
	Liver diseases N% 24 (6.2%),	1(4.2%)	7 (29.2%)	8 (33.3%)	8 (33.3%)
	HIV/AIDS N% 16 (4.1%),	3 (18.8%)	3 (18.8%)	3 (18.8%)	7(43.8%)
	Others N% 17 (4.4%)	2(11.8%)	9(52.9%)	0(0%)	6 (35.3%)

* Cell total

**Pre-hospital characteristics**: Most of the participants were self-referral 118 (30.4%) followed by public hospitals, 87 (22.4%), health center, 54 (13.9%), unknown source of referral accounts about 52 (13.4%), SPHMMC- outpatient 42 (10.8%), private Hospitals referred 25 cases (6.4%), SPHMMC staff family and relatives 8 (2.1%) and least source of referral being private clinic 2 cases (0.5%). Majority of critically sick patients were transported by taxi (23.2%) followed by ambulance (19.1%) and private car (17.5) (Table 1).

**Clinical characteristics of participants**: The burden of non-traumatic acute illness as ED working diagnosis were the most common 351 cases (90.7%). There were 35 (9.0%) participants kept in ED with unclassified acute illness (trauma or non-traumatic). The magnitude of trauma related cases was negligible 2(0.3%).

Among non-traumatic medical emergencies, the most common was sepsis and septic shock 65(16.8%) followed by respiratory illness 50 (12.9%), renal diseases 44 (11.3%), CVD 43 (11.1%) CNS diseases 41(10.6%), liver diseases 27 (7.0%), oncologic emergencies 24 (6.2%), UGIB 12 (3.1%), severe anemia and acute complications of DM 10 cases (2.6%) each, unspecified medical emergencies 14 (3.6%), other medical acute illnesses 11 cases (2.8%) were like other infection, psychiatric illness epilepsy, etc.

Most of participants 279 (79.9%) had comorbid illness and only 109 (28.1%) served in SPHMMCED have been documented as having no comorbid illness. Of those having comorbid illness, the most common comorbid illness renal related diseases (chronic kidney diseases, end stage renal diseases etc.) and malignancy accounts about 12.6% (49 cases) each followed by hypertension 43(11.1%), respiratory illness (asthma, chronic obstructive pulmonary disease etc.) 29 (7.5%), other cardiovascular illness (heart frailer, etc.) 27 (7.0%), DM and its complications 25 (6.4%), liver diseases 24 (6.2%), HIV/AIDS, 16 (4.1%), and others (CNS, psychiatric illness, other infections etc.) 17 (4.4%) (Table 1).

**Health care financing details (method of payment):**

Of registered specified method of payment in ED for study participants, out of pocket payment 139(35.8%) was the most common followed by CBHI 60 (15.5%) and fee waived by government 53 (13.7%).

**Deleted: <*object*>**

Out-of-pocket payment for health care service in critically sick SPHMMC clients was high across all age groups in comparison to other specified methods. Participant service fee waived by government and paid via CBHI were comparable among genders, but out of pocket payment is relatively higher in female sub-groups. The magnitude of out-of-pocket payment mechanisms were uniformly higher across all registered address regions, source of referrals, mode of arrival to ED and in those having comorbid conditions.

## Discussion

This sub-section, discusses major findings of the study, its impacts and implications, and comparison with the findings of other available literature. Over 30 months SPHMMC- ED served 22, 982 clients with a mean age of 49.84 ± 18.21 years. The most common participants being in the age group between 30-60 (47.7%). Male to female ratio of the participants was 1.3:1. This finding is consistent with two studies done in Black lion Hospital, an affiliate of Addis Ababa University (17, 18).

The majority of the participants came from Addis Ababa and Oromia region (84.2%), the least participants were from Gambella region (0.3%). Even though as a tertiary hospital, SPHMMC is expected to provide services to clients from the whole regions of Ethiopia, the distribution of participants' regions was centered over two central regions of the country. It could be due to an acute condition being cared for in their vicinity or dying shortly, or it could be associated with limited emergency medical services. Further multicentre study is required to explain this finding.

The burden of non-traumatic acute illness as ED working diagnosis were the most common (90.7%). There were (9.0%) participants kept in ED with unclassified acute illness (trauma or nontraumatic). The magnitude of trauma related cases was negligible 2(0.3%). Among non-traumatic medical emergencies, the top three were sepsis and septic shock (16.8%) followed by respiratory illness (12.9%), renal diseases 44 (11.3%). Most of participants (79.9%) had comorbid illness, and of those having comorbid illness, the most common comorbid illness renal related diseases and malignancy accounts about (12.6%) each followed by hypertension (11.1%).

Of specified methods of payment in ED for study participants, out of pocket payment 139(35.8%) is the most common followed by CBHI 60 (15.5%) and fee waived by government 53 (13.7%). This finding is consistent with the Debie et al (14) and lower by more than half than findings from Hunchak et al study (16). This could be probably due to different health financing related reforms in Ethiopia like implementation CBHI, which has been more enforced in recent years. The primary aim of HCF reforms like implementing CBHI is to reduce the burden of out-of-pocket expenditure related hardship and enhance financial protection, equity and sustainability. The socio-economic impact of OOP is significant in LMIC, where it can force the payer to impoverishment in 5% of global population (18, 19). Out of pocket payment was relatively higher in female sub-groups. Its impact on vulnerable sections of the community like females and patients with comorbid conditions would be worse as per finding of study by (Obse and Ataguba BMC International Health and Human Rights) (19). Special attention should be given to those vulnerable towards poverty.

Out of pocket payment for health care service in critically sick SPHMMC clients remained a major method of expenditure across all age groups in comparison to other specified methods. The magnitude of out-of-pocket payment mechanisms were uniformly higher across all registered address regions, source of referrals, mode of arrival to ED and in those having comorbid conditions. OOP seems to be a global public health challenge in general and more specifically in sub–Saharan Africa where it remained above 35% (10), this study finding has consistency with WHO global health expenditure database (10).

Despite the fact that this is a cross-sectional single-center study, which makes generalization difficult, it provides evidence for researchers, policy makers and institutional leaders to focus on the highlighted shortcomings.

As conclusion; the means of expenditure for health care in study participants were CBHI accounting in about 15.5% and government waived fee in about 13.7% and out of pocket in about 35.8%. A major method that was consistently greater across all age groups, both genders, address regions, referral sources, modes of arrival into ED, and comorbid conditions was out-of-pocket spending for health care As OOP means of payment in acutely ill and injured is highest, and that may result in impoverishment on payer, negatively affect financial protection, equity and access to health care. Hence the responsible body should intervene to lower this impact in general. There has to be some means that reduce the impact of OOP on vulnerable communities like women and those with comorbid conditions. Further prospective, multi-center study on burden and predictors of out-of-pocket payment recommended to fully understand in detail.
